# New insights into the Tat protein transport cycle from characterizing the assembled Tat translocon

**DOI:** 10.1111/mmi.14984

**Published:** 2022-10-05

**Authors:** Felicity Alcock, Ben C. Berks

**Affiliations:** ^1^ Department of Biochemistry University of Oxford Oxford UK; ^2^ Microbes in Health and Disease Theme, Newcastle University Biosciences Institute Newcastle University Newcastle upon Tyne UK

**Keywords:** cell membrane, protein transport, protonmotive force, twin‐arginine translocation system

## Abstract

The twin‐arginine protein translocation (Tat) system transports folded proteins across the bacterial cytoplasmic membrane and the thylakoid membrane of chloroplasts. The Tat translocation site is transiently assembled by the recruitment of multiple TatA proteins to a substrate‐activated TatBC receptor complex in a process requiring the protonmotive force. The ephemeral nature of the Tat translocation site has so far precluded its isolation. We now report that detergent solubilization of membranes during active transport allows the recovery of receptor complexes that are associated with elevated levels of TatA. We apply this biochemical analysis in combination with live cell fluorescence imaging to Tat systems trapped in the assembled state. We resolve sub‐steps in the Tat translocation cycle and infer that TatA assembly precedes the functional interaction of TatA with a polar cluster site on TatC. We observe that dissipation of the protonmotive force releases TatA oligomers from the assembled translocation site demonstrating that the stability of the TatA oligomer does not depend on binding to the receptor complex and implying that the TatA oligomer is assembled at the periphery of the receptor complex. This work provides new insight into the Tat transport cycle and advances efforts to isolate the active Tat translocon.

## Graphical abstract

The Tat translocon transports folded proteins via a unique and enigmatic mechanism. The translocon assembles transiently, only in the presence of a substrate, a protonmotive force, and only at native expression levels, and for this reason is exceedingly difficult to access experimentally. The recovery of a partially assembled Tat apparatus described here validates previous work with fluorescent fusion proteins, provides new insight into the transport cycle, and is a significant advance in our efforts to isolate the active translocon.

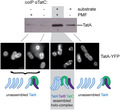

## 1 INTRODUCTION

The twin‐arginine translocation (Tat) system (Berks, 2015; Cline, 2015; Hamsanathan & Musser, 2018; Palmer & Stansfeld, 2020) is one of two general protein export pathways found in the cytoplasmic membrane of prokaryotes (Palmer & Berks, 2012) and is evolutionarily conserved in the chloroplast thylakoid membrane and the inner membrane of some mitochondria (Burger et al., 2013; Carrie et al., 2016; Celedon & Cline, 2013; Petru et al., 2018; Schafer et al., 2020). The protein substrates of the Tat system fold before transport yet are thought to be translocated without compromising the ionic integrity of these tightly sealed membrane systems (Teter & Theg, 1998). How this is achieved mechanistically is unclear. However, one potential contributory factor is that the active Tat translocation site is only assembled in the presence of substrate proteins.

Tat transport in the bacterium *Escherichia coli* is mediated by the three integral membrane proteins TatA, TatB, and TatC. *E. coli* also possesses a TatA paralog, TatE. However, TatE is expressed at much lower levels than TatA and is not required for Tat transport when TatA is present (Jack et al., 2001; Sargent et al., 1998). Substrate proteins are targeted to the Tat system by N‐terminal signal peptides containing an invariant twin‐arginine motif (Berks, 1996; Chaddock et al., 1995; Palmer & Stansfeld, 2020; Stanley et al., 2000). The Tat signal peptide is recognized by a receptor complex containing the TatB and TatC proteins together with a small proportion of the TatA proteins present in the membrane (Alami et al., 2003; Alcock et al., 2016; Cline & Mori, 2001). The exact polypeptide composition of this TatABC complex is still unclear, though it is known to contain multiple copies of each of the three constituent components at an apparently equimolar ratio (Alcock et al., 2016; Bolhuis et al., 2001; Zoufaly et al., 2012). Following initial substrate binding, the signal peptide is inserted more deeply in the membrane (Gerard & Cline, 2007; Hamsanathan et al., 2017) and the receptor complex recruits many further TatA protomers from a pool in the membrane to form the active translocation site (Alcock et al., 2013; Dabney‐Smith et al., 2006; Rose et al., 2013). Models of the receptor complex place TatBC protomers in a ring, enclosing an internal cavity where the substrate signal peptide is expected to dock (Alcock et al., 2016; Blummel et al., 2015; Cline, 2015; Habersetzer et al., 2017). Different mechanistic models suggest that the recruited TatA molecules either oligomerize on the periphery of the receptor complex or accumulate within its interior cavity (Blummel et al., 2015; Cline, 2015; Frobel et al., 2019; Tarry et al., 2009). Transport of the substrate across the membrane is assumed to be mediated primarily by the TatA oligomer within the translocation complex (Cline, 2015). The TatA oligomer disassembles once transport is complete (Mori & Cline, 2002).

Tat transport requires the transmembrane protonmotive force (PMF) (Bageshwar & Musser, 2007; Braun et al., 2007; Mould & Robinson, 1991; Yahr & Wickner, 2001). The PMF is necessary for the TatA oligomerization step of the transport cycle (Alami et al., 2003; Alcock et al., 2013; Cline & Mori, 2001; Mori & Cline, 2002; Rose et al., 2013). What mechanistic role the PMF plays in this step and whether the PMF acts elsewhere in the Tat cycle are uncertain. It has recently been argued that the PMF does not do active work to drive Tat transport, but rather that the PMF‐dependence of Tat transport reflects the fact that the Tat components have evolved to work in a milieu where the PMF is an integral part of their membrane environment (Hamsanathan & Musser, 2018). More specifically, it was proposed that the PMF is necessary for the receptor complex to maintain a conformation that is competent for TatA oligomerization.

The structures of the individual Tat components have been determined (Hu et al., 2010; Ramasamy et al., 2013; Rodriguez et al., 2013; Rollauer et al., 2012; Zhang et al., 2014) and a detailed molecular model for the TatBC receptor complex has been proposed based on sequence co‐evolution analysis, molecular modeling, and biochemical data (Alcock et al., 2016). In this receptor complex model, TatB binds to an intramembrane site on TatC, which includes a cluster of polar amino acids. At some point following substrate activation of the receptor complex, TatA transiently replaces TatB at this polar cluster site (Alcock et al., 2016; Habersetzer et al., 2017).

In spite of these structural advances, elucidating the molecular basis of Tat transport requires knowledge of the structure of the fully assembled active Tat translocation site. Isolation of this transiently formed complex is an exceedingly challenging task, and it has been assumed that the assembled translocon is too ephemeral and unstable to be purified. Here, we show that TatABC complexes retaining elevated levels of TatA are obtained if detergent solubilization of assembled Tat translocon complexes is carried out directly from energized membranes. This approach overcomes a key roadblock in the isolation of the assembled Tat translocation site. Combining this methodology with live cell imaging experiments provides new insight into the Tat translocation cycle and shows that the receptor complex is not essential for TatA oligomer stability once the oligomer has formed.

## 2 RESULTS

### 2.1 Strategy for the solubilization of assembled Tat translocation sites

Many years of biochemical analysis of the Tat system has led to the view that the assembled translocation site is formed too transiently to be successfully extracted with detergents from its native membrane environment. Nevertheless, imaging studies of *E. coli* cells have shown that if Tat pathway flux is maximized by the provision of saturating levels of substrate proteins then the majority of the TatA molecules in the cell are found in the assembled state (Alcock et al., 2013; Rose et al., 2013). This suggests that the failure to isolate assembled Tat translocons might not be a consequence of their short lifetime or low steady state abundance but, instead, could be due to translocon disassembly during preparation of the membrane fraction used for the detergent solubilization step. If this is the case, then we reasoned that it would be possible to trap assembled translocons if the solubilizing detergent was added directly to cells engaged in active Tat transport. A practical issue in implementing this approach is that the outer membrane of *E. coli* acts as a barrier that impedes detergent ingress to the cell (Nikaido, 2003). Consequently, we chose to solubilize spheroplasted cells in which the outer membrane has been permeabilised by EDTA treatment and the cell wall (to which the outer membrane is anchored) has been digested away with lysozyme. Since Tat transport is an exclusively inner membrane process, spheroplasts remain able to carry out Tat transport (Masui et al., 1994). In our experiments, we overproduce a Tat substrate protein in cells possessing the endogenous Tat proteins. This arrangement saturates the Tat pathway with substrate, ensuring that the Tat components are in the assembled state (Alcock et al., 2013). Working with the native Tat system also avoids the perturbations of Tat protein interactions that are an issue when Tat components are overproduced (Alcock et al., 2016).

Our approach differs from previous purifications of bacterial TatABC complexes which all start from de‐energized membrane preparations obtained by the mechanical disruption of either cells or spheroplasts, and which are carried out in the absence of overproduced substrate proteins (e.g. Bolhuis et al., 2001; de Leeuw et al., 2002). In contrast to our new method, these earlier purification methods fail to provide the PMF and substrate triggering needed for Tat translocon assembly. Consistent with this, the TatABC complexes purified by these methods do not contain a molar excess of TatA over TatB and TatC (Bolhuis et al., 2001).

### 2.2 Elevated levels of TatA in TatABC complexes solubilized from energized membranes

**FIGURE 1:**
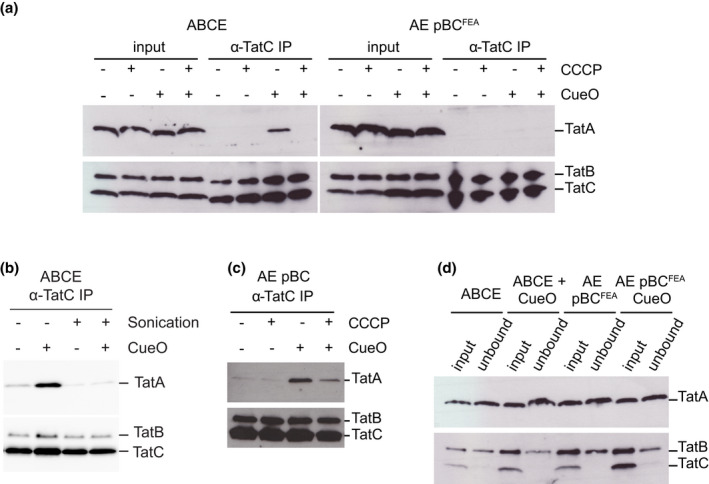
Substrate‐induced association of TatA with TatC is maintained upon detergent solubilization of energized spheroplasts. (a–c) Digitionin‐solubilized spheroplasts of the indicated strains were immunoprecipitated with antibodies against TatC and then immunoblotted with either a combination of TatB and TatC antibodies (lower panels) or with TatA antibodies (upper panels). Where indicated the strains were induced for high level production of the plasmid‐encoded Tat substrate protein CueO. Where indicated strains were treated with CCCP to dissipate the PMF before addition of detergent. The TatC^FEA^ variant is blocked in substrate interactions. (a) The immunoprecipitates (‘α‐TatC IP’ lanes) are compared with the spheroplast extract before the immunoprecipitation step (‘input’ lanes) corresponding to 1% of the input for TatA or 25% for TatB and TatC. (b) Where indicated, strains were sonicated prior to digitonin‐solubilization. (d) Comparison between the soluble extract used for the immunoprecipitation (‘input’ lanes) and the material that remains in solution following the immunoprecipitation step (‘unbound’ lanes). Strains have a wild‐type Tat system (ABCE) or are Δ*tatBC* but complemented with a plasmid expressing either *tatBC* or *tatBC* ^*FEA*^ at native levels (AE pBC and AE pBC^FEA^ respectively). [Correction added on 18 December 2022,after first online publication: In Figure 1, the labels ‘1% input’ have been changed to ‘input’ in this version.]

To put our translocon solubilization strategy into practice, we generated spheroplasts from wild‐type *E. coli* cells (termed strain ‘ABCE’ to indicate that it expresses native levels of TatA, TatB, TatC, and TatE) overproducing the native Tat substrate protein CueO from an IPTG‐inducible promoter. The detergent digitonin was chosen to solubilize the spheroplast membranes because it is known to maintain the integrity of the TatBC receptor complex that lies at the core of the assembled translocon (Bolhuis et al., 2001). The presence of TatABC complexes in the detergent extracts was assessed by monitoring the amount of TatA that co‐immunoprecipitates with TatC. Although the resting TatABC receptor complex contains a small amount of constitutively bound TatA (Alcock et al., 2016), our assay was optimized to detect the higher amounts of TatA we expected to find in the substrate‐activated translocons. Consequently, in our assay negligible amounts of TatA were detected in complex with TatC in the absence of the overproduced substrate protein (Figure 1a). However, when spheroplasts were prepared from a strain overproducing CueO, there was clear association of TatA with TatC (Figure 1a), and this association was abolished if the PMF was collapsed by treating the spheroplasts with the protonophore carbonyl cyanide‐*m*‐chlorophenyl hydrazine (CCCP) prior to detergent addition. Thus, our spheroplast solubilization approach is able to detect the substrate‐ and PMF‐dependent pattern of TatA association with TatC that characterizes the operation of the native Tat pathway. As expected, TatB was co‐immunoprecipitated with TatC irrespective of the presence of overproduced substrate protein or the PMF (Figure 1a).

To assess the improvement of our approach over earlier purification methods, we directly compared samples prepared by our method using intact spheroplasts with equivalent samples prepared from sonicated spheroplasts as employed in previous purification studies (Bolhuis et al., 2001). Substrate‐induced TatA recruitment was only observed for the intact spheroplasts (Figure 1b), demonstrating that only our new method is capturing assembly events.

We repeated our intact spheroplast solubilization approach using spheroplasts expressing the ‘FEA’ (F94A, E103A) variant of TatC (strain ‘AE pBC^FEA^’). This variant is blocked in Tat signal peptide binding and is therefore unable to undergo substrate‐induced TatA oligomerization (Alcock et al., 2013). Using these spheroplasts, we no longer observed the enhanced binding of TatA with TatC in the presence of overproduced substrate protein (Figure 1a), again mirroring the behavior of the physiological Tat pathway. For reasons of technical convenience, as in previous studies (Alcock et al., 2013, 2016), the TatC^FEA^ variant was co‐produced with TatB from a plasmid (pBC^FEA^) rather than from the chromosomal *tat* locus. Both pBC^FEA^ and the parental TatBC‐producing plasmid (pBC) produce TatB and TatC at levels comparable to the endogenous proteins (Alcock et al., 2016) and control experiments confirm that pBC is able to support substrate‐ and PMF‐dependent association of TatA with TatC (Figure 1c).

In our solubilized spheroplast experiments, the TatC immunoprecipitation reactions removed all TatC molecules from the detergent extracts, together with the bulk of the TatB molecules (Figure 1d). However, under the condition where we see the substrate‐ and PMF‐dependent co‐immunoprecipitation of TatA with TatC, there is no discernible depletion of TatA from the extract (Figure 1d). Thus, only a small proportion of the total TatA pool is recovered in complex with TatC. The same inference can be drawn by comparing the amount of TatA present in the soluble extracts with the amount of TatA recovered by co‐immunoprecipitation with TatC (Figure 1a). Given the expectation from fluorescence imaging experiments that most of the TatA molecules in the starting cells will be found in assembled translocons (Alcock et al., 2013), our observations suggest that only a small proportion of the Tat translocons survive the solubilization and isolation procedure or, alternatively, only a small proportion of the TatA molecules in each oligomer remain bound to TatC.

To summarize, direct solubilization of energized spheroplasts qualitatively reproduces the pattern of Tat translocon assembly observed in vivo, but the total amount of TatA recovered with TatBC is less than expected from the high degree of assembled complexes present in the starting material. Thus, while this strategy for the preparation of an assembled Tat translocon is a clear advance on previous isolation efforts, further methodological developments are required to better maintain the integrity of the translocon during detergent extraction. In the next section, we explore whether the required stabilization of the Tat translocation site to solubilization can be achieved by locking the translocon in the assembled state.

### 2.3 Characterization of strains that trap assembled TatA oligomers

A number of Tat variants have been reported to stabilize TatA in an assembled state (Alcock et al., 2013, 2017; Huang et al., 2017; Leake et al., 2008). Most of these variants are transport‐inactive and are assumed to act by blocking Tat transport between the translocon assembly and disassembly steps of the transport cycle (Alcock et al., 2017). By contrast, a transport‐permissive TatB F13Y variant induces constitutive translocon assembly by mimicking the assembly triggering effects of substrate binding to the TatBC complex (Huang et al., 2017). Given the apparent stabilization of the assembled Tat translocation site in strains expressing these Tat variants, we decided to investigate whether the variants would exhibit enhanced TatA‐TatC interactions in our direct solubilization protocol. Before undertaking this analysis, we first needed to establish that the variants exhibited the expected TatA assembly and Tat transport phenotypes in the standardized experimental conditions and genetic backgrounds used in this study. These data were subsequently used to allow direct comparison of the TatA assembly phenotypes determined by the new spheroplast solubilization approach with those determined by the established live cell imaging method detailed below. As part of this analysis, we surveyed additional transport‐inactive Tat variants in an attempt to discover further assembly‐trapped mutants.

**FIGURE 2:**
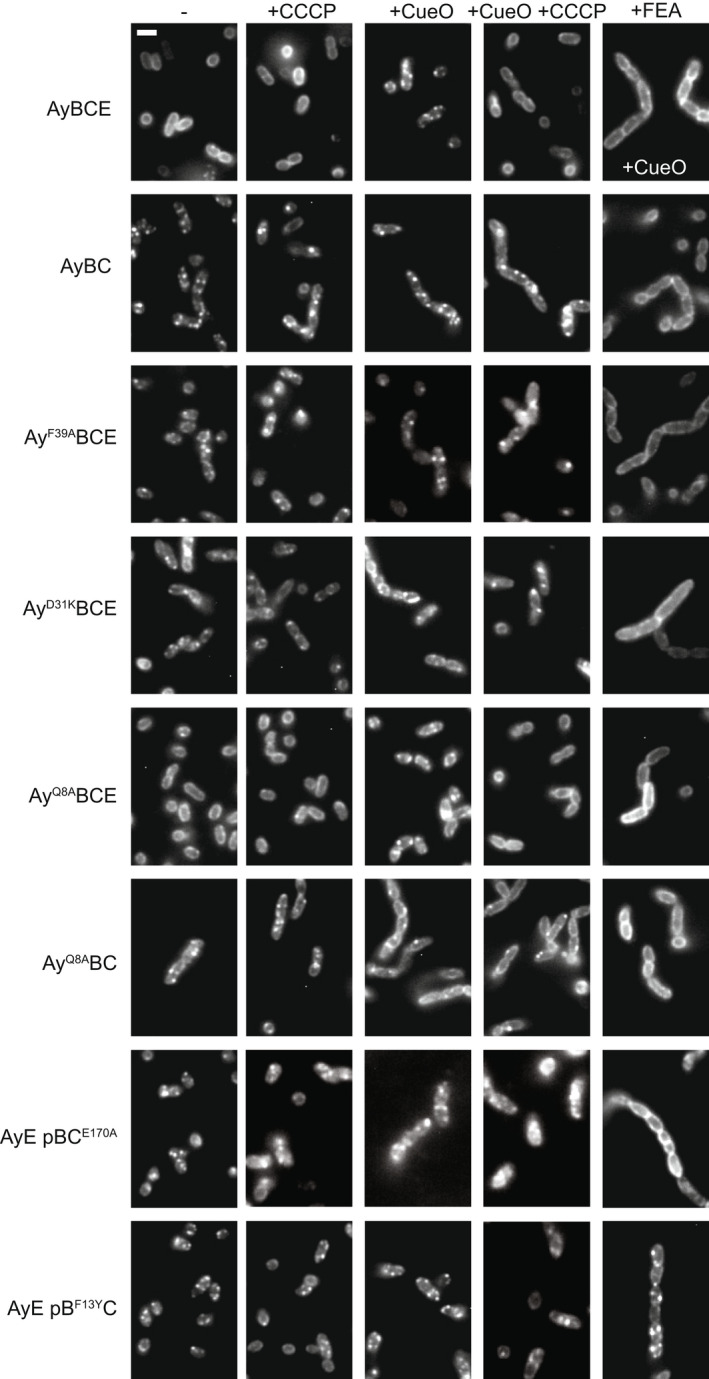
The effects of amino acid substitutions in Tat components on the substrate‐induced oligomerization of TatA‐YFP in live cells. Representative fluorescence images of TatA‐YFP in living cells. The strain used is indicated to the left of each row. Where indicated, strains were induced for high level production of the Tat substrate protein CueO (+CueO columns, and the final panel in the top row). Where specified, cells were treated with CCCP prior to imaging to dissipate the PMF (+CCCP columns). The TatC^FEA^ variant was used to test the effect of blocking substrate interactions in the absence of an overproduced substrate (+FEA column). To achieve this, the strains in +FEA column contain a chromosomal Δ*tatBC* deletion and a plasmid encoding TatB and TatC^FEA^ (pBC^FEA^). Scale bar = 2 μM (shown in top left panel). Strains are named for the Tat proteins they produce (Ay = TatA‐YFP, B = TatB, C = TatC, E = TatE) with amino acid substitutions given as superscripts to the protein in which they are located and ‘p’ indicating that the proteins that follow are expressed at native levels from a plasmid. [Correction added on 18 December 2022, after first online publication: Figure 2 inadvertently included duplicate images and has been updated in this version.]

The TatA assembly behavior of the variants in live cells was assessed using a TatA‐YFP reporter (Alcock et al., 2013), which we represent by the shorthand ‘Ay’ when listing the Tat components present in the strains. It is important to note at this point that the TatA‐YFP fusion is itself transport‐inactive in cells lacking the TatA paralog TatE and accumulates in the assembled state (in other words the transport defective phenotype of *tatA*‐*yfp* is conditional on a Δ*tatE* background) (Alcock et al., 2013; Leake et al., 2008). For this reason, we also included a strain without TatE (strain AyBC) in the collection of assembly locked mutants under analysis. Substrate‐induced TatA oligomerization in the parental strain is manifested as a change from disperse peripheral fluorescence in the absence of overproduced substrate, to multiple bright fluorescent foci when a substrate protein is overproduced (strain AyBCE in Figure 2) (Alcock et al., 2013). If a mutation blocks Tat translocon assembly, then the peripheral halo of TatA‐YFP fluorescence remains unaltered after substrate overproduction (as observed, for example, by introducing the FEA mutations into AyBCE in the presence of CueO overproduction, Figure 2). However, in strains in which TatA‐YFP is stabilized in the assembled state, the fluorescent foci are present even without substrate overproduction (in Figure 2 compare –CueO and + CueO lanes for strains AyBC, Ay^F39A^BCE, Ay^D31K^BCE, and AyE pB^F13Y^C). For those of these strains blocked in substrate transport (strains AyBC, Ay^F39A^BCE, and Ay^D31K^BCE), this behavior arises because even endogenous levels of substrate proteins are sufficient to achieve TatA‐YFP oligomerization when the substrate is not being released through transport. Consistent with this explanation, substitutions that preclude substrate interactions with the TatBC complex prevent TatA‐YFP foci forming in these strains (+FEA panels in Figure 2). By contrast, in strain AyE pB^F13Y^C assembly of TatA‐YFP is substrate‐independent because this mutant is constitutively activated for translocon assembly. Consequently, the TatA‐YFP foci in this strain are insensitive to substitutions that prevent substrate binding by TatBC (+FEA panel in Figure 2). Importantly, and characteristically, the stabilized TatA‐YFP foci present in the strains of interest persist even after the PMF has been dissipated with a protonophore (+ CueO + CCCP panels in Figure 2). This observation demonstrates that the assembled state of TatA that has been trapped in these mutants does not require continuing energization for its stability (Alcock et al., 2013). In addition to the previously identified substitutions, we found that a transport‐inactivating TatC E170A substitution also resulted in protonophore‐resistant oligomerization of TatA‐YFP in the absence of substrate overproduction (Figure 2, strain AyE pBC^E170A^). The results of the fluorescence imaging experiments are summarized in Table 1.

**TABLE 1:** Summary of TatA oligomerization behavior observed by TatC co‐immunoprecipitation and fluorescence imaging

Strain	TatA oligomers observed					Tat activity		
		−	+CCCP	+CueO	+CueO +CCCP	Cell chaining^a^	SDS resistance^b^	CueO export^c^
ABCE	IP	−	−	+	−	S	100	+
AyBCE	IP	−	−	+	−	S	84	+
	Fluorescence	−	−	+	−			
ABC	IP	−	−	+	−	S	85	+
AyBC	IP	+	−	+	−	SC	25	−
	Fluorescence	+	+	+	+			
A^F39A^BC	IP	+	−	+	−	C	24	−
A^F39A^BCE	IP	+	−	+	−	SC	42	−
A_y_^F39A^BCE	IP	+	−	+	−	SC	32	−
	Fluorescence	+	+	+	+			
A^D31K^BC	IP	+	−	+	−	C	6	−
A^D31K^BCE	IP	+	−	+	−	SC	10	−
A_y_^D31K^BCE	IP	+	−	+	−	SC	15	−
	fluorescence	+	+	+	+			
A^Q8A^BC	IP	+	−	+	−	SC	16	−
A^Q8A^BCE	IP	−	−	+	−	S	77	+
A_y_^Q8A^BCE	IP	−	−	+	−	S	75	+
	Fluorescence	−	−	+	−			
AE pBC^E170A^	IP	+	+	+	+	S	93	−
AyE pBC^E170A^	IP	+	+	+	+	S	66	−
	Fluorescence	+	+	+	+			
AE pB^F13Y^C	IP	+	−	+	−	S	94	+
AyE pB^F13Y^C	IP	+	−	+	−	SC	27	−
	Fluorescence	+	+	+	+			

^a^ S = single cells (Tat^+^), SC = single cells and short chains (impaired Tat activity), C = fully chained (Tat^−^).

^b^ + = growth in the presence of 2% SDS, percentage relative to wild‐type.

^c^ + = export detected, − = no export detected.

The Tat transport activity of the mutants of interest was systematically characterized by standardized assays both for strains expressing the TatA‐YFP fusion and for strains expressing the wild‐type TatA protein. In the latter case we additionally assessed the effect of removing the TatA paralog TatE such that transport activity in the strain is fully TatA‐dependent. Three assays with sensitivity to different ranges of Tat activity were used. In the first assay, the Tat substrate protein CueO was over‐produced and subcellular fractionation used to determine the amount of this protein reaching the periplasm. Under these conditions of substrate saturation, the export of CueO is proportional to the transport capacity of the Tat pathway. However, as this assay is not sufficiently sensitive to determine whether very low levels of Tat activity are still present, we also examined the mutant strains for two phenotypes that are only observed in cells with an almost complete loss of Tat function and which arise from a failure to export Tat‐dependent amidases: cell chaining and sensitivity to the detergent SDS (Ize et al., 2003).

**FIGURE 3:**
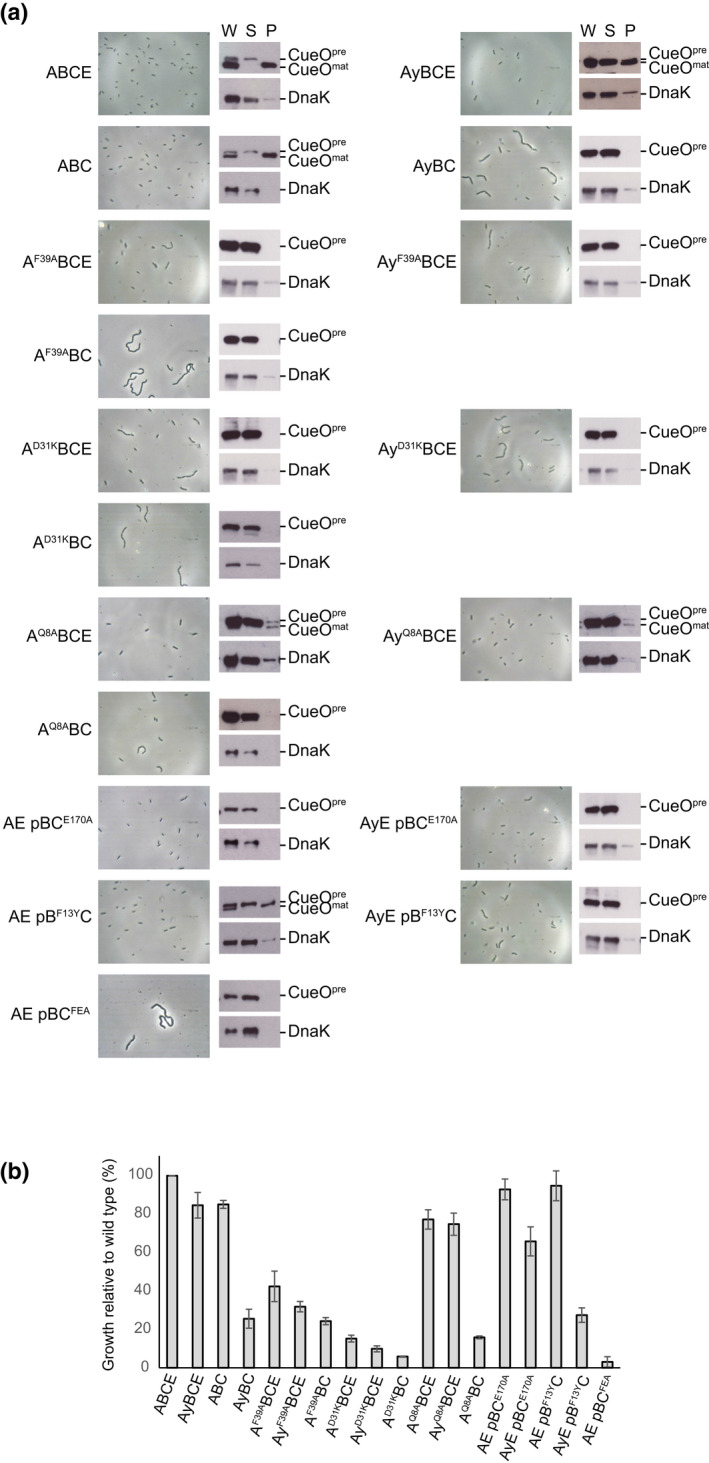
The effects on Tat transport activity of amino acid substitutions in the Tat components. Strains contained the indicated amino acid substitutions in chromosomally‐encoded TatA or plasmid‐encoded TatB or TatC. (a) The left hand panel in each pair shows a phase contrast image of the strain. The right hand panel in each pair assesses the transport activity of the strain when overproducing the Tat substrate CueO. Whole cell (W), spheroplast (S) and periplasm (P) fractions were subject to immunoblotting with antibodies against CueO or the cytoplasmic marker protein DnaK. ‘CueOmat’ is the transported form of CueO from which the signal peptide has been removed and ‘CueOpre’ the precursor protein. The TatC^FEA^ variant is blocked in substrate interactions and acts as a negative control for transport. (b) Strains were grown in LB + 4% SDS and the quotient of the OD600 with/without SDS is shown as a percentage of the wild‐type. Error bars correspond to the SEM (*n* = 3). Strains are named for the Tat proteins they produce (A = TatA, B = TatB, C = TatC, E = TatE, Ay = TatA‐YFP) with amino acid substitutions given as superscripts to the protein in which they are located and ‘p’ indicating that the proteins that follow are expressed at native levels from a plasmid.

As expected, all the mutant strains except *tatB* ^*F13Y*^ had severely compromised CueO export activity (Figure 3a, data summarized in Table 1). Many strains exhibited poor SDS‐resistance and partial cell chaining, though only the TatA^D31K^ substitution (in the absence of TatE) was found to be completely Tat deficient in both the cell‐chaining and SDS‐sensitivity assays (Figure 3a,b data summarized in Table 1).

**FIGURE 4:**
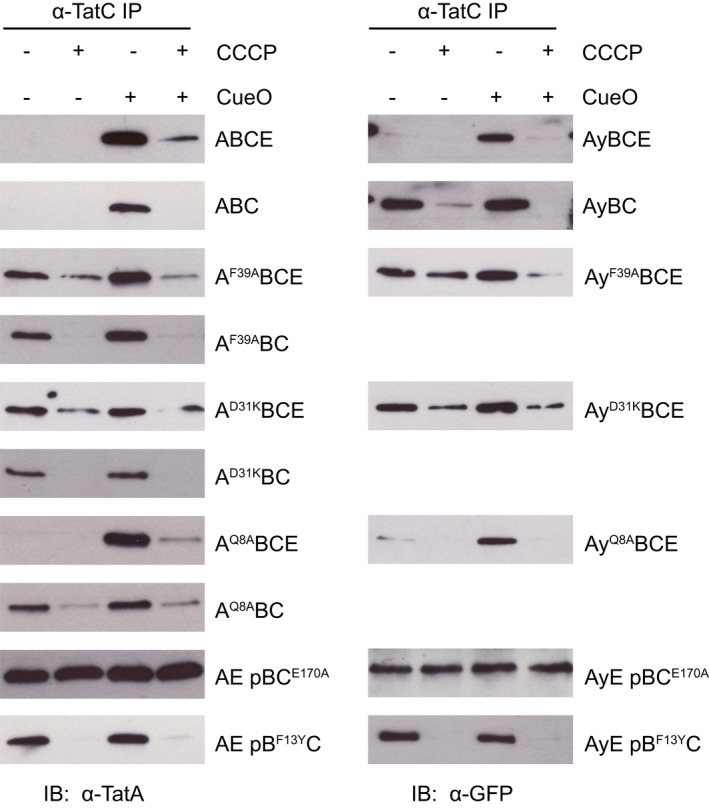
The effects of amino acid substitutions in Tat components on the association of TatA with TatC in detergent extracts. Digitionin‐solubilized spheroplasts of the indicated strains were immunoprecipitated with antibodies against TatC (‘α‐TatC IP’) and then immunoblotted with TatA antibodies (‘IB: α‐TatA’) or GFP antibodies (to detect YFP) (‘IB: α‐GFP’). Where indicated, strains were induced for high level production of the plasmid‐encoded Tat substrate protein CueO. Where specified, cells were treated with CCCP to dissipate the PMF before addition of detergent. Strains are named for the Tat proteins they produce (A = TatA, B = TatB, C = TatC, E = TatE, Ay = TatA‐YFP) with amino acid substitutions given as superscripts to the protein in which they are located and ‘p’ indicating that the proteins that follow are expressed at native levels from a plasmid.

In an earlier work, it was concluded that the *tatB* ^*F13Y*^ mutation drives constitutive translocon complex assembly based on the observation that it induces substrate‐independent oligomerisation of TatA‐YFP (Huang et al., 2017). However, we observe here that introducing the *tatB* ^*F13Y*^ allele into a strain expressing TatA‐YFP results in the cells losing the ability to transport CueO (Figure 3a, compare the high levels of CueO transport in strains AE pB^F13Y^C and AyBCE with the absence of detectable CueO transport in strain AyE pB^F13Y^C). This observation raises the question as to whether it is really the *tatB* ^*F13Y*^ mutation that drives TatA‐YFP oligomerization or whether the oligomerization is a result of inactivating the Tat system as in other strains exhibiting stable TatA‐YFP assembly. However, TatA‐YFP assembly still occurs when substrate binding to the TatB^F13Y^C complex is blocked (Figure 2, AyE pB^F13Y^C + FEA panel), and thus is substrate‐independent, whereas in the transport‐inactive Tat variants substrate binding is required for TatA‐YFP oligomerization. Furthermore, we show below by co‐immunoprecipitation that the TatB^F13Y^ variant still drives assembly of TatA on to TatC when the YFP domain is absent from TatA (Figure 4). Thus, it remains valid to infer that the TatB F13Y substitution causes TatA oligomerization through a substrate‐independent mechanism.

### 2.4 Assessment of TatA‐TatC association in strains with trapped TatA oligomers

We show above that application of our direct solubilization method to the native Tat system is able to capture the substrate‐ and PMF‐dependent assembly of the Tat translocation site that is observed by in‐cell fluorescence imaging of a TatA‐YFP fusion. To directly test the equivalence of the two analytical methods we assessed the behavior of the TatA‐YFP fusion in the direct solubilization assay. Recruitment of the TatA‐YFP fusion to TatC was, as in the imaging analysis, dependent on substrate overproduction and spheroplast energization (Figure 4 strain AyBCE). Thus, the direct solubilization and fluorescence imaging methods are directly comparable. Importantly, this experiment shows that for an active Tat system containing TatE, the TatA assembly behavior measured by the direct solubilization method is unaffected by the presence of a YFP fusion domain on TatA (Figure 4 compare strains ABCE and AyBCE).

Having confirmed that the set of variant Tat systems stably assemble TatA‐YFP oligomers under our experimental conditions, we applied our direct solubilization protocol to assess whether these variants also increased the amount of TatA associated with TatC in detergent solution.

For the TatC^E170A^ variant, the solubilization and imaging data were in full agreement. For this variant, elevated levels of TatA co‐purified with TatC and assembled TatA‐YFP foci were present, regardless of whether substrate protein was overproduced or the PMF was present (Figures 2 and 4 strain AyE pBC^E170A^).

The TatA^F39A^, TatA^D31K^ and TatB^F13Y^ variants also exhibited increased TatA co‐purification with TatC in the absence of substrate overproduction, as expected from the imaging results (Figure 4). However, for these variants, the addition of protonophore either eliminated (TatA^F39A^ and TatA^D31K^ in the absence of TatE, or TatB^F13Y^ in the presence of TatE) or heavily reduced (TatA^F39A^ and TatA^D31K^ in the presence of TatE) the TatA‐TatC interaction, both in the presence and absence of overproduced substrate (Figure 4). This is in stark contrast to the imaging experiments where the oligomerization of TatA was maintained upon protonophore addition (Figure 2). We can exclude the possibility that this difference in behavior is due to the presence of the YFP domain that is fused to TatA in the imaging experiments because the results of the solubilization experiments were the same whether the YFP reporter was present on TatA or not (Figure 4; note that because the TatA‐YFP fusion is inactive in the absence of TatE, we only show solubilization experiments for TatA‐YFP‐containing strains that also express TatE). Instead, these differences can be interpreted as reflecting the different aspects of Tat translocon assembly that are characterized by the two analytical methods: TatA oligomeric state in the case of the imaging assay and TatA‐TatC associations in the case of the direct solubilization approach. Thus, in these variants the TatA‐TatC interaction is fully labile to protonophore treatment whilst TatA oligomer stability is apparently unaffected.

In the TatA‐YFP imaging approach the presence of the TatA paralogue TatE is required to realize the transport activity of TatA‐YFP (Alcock et al., 2013). By contrast, because the direct solubilization assay does not require a YFP domain on TatA: this allows us to assess the effects of substitutions in TatA in the absence of TatE, and thus without the confounding presence of this TatA paralogue. This feature of the direct solubilization assay prompted us to revisit the effects on TatA assembly of substituting Gln8, a residue which is known to be important for Tat transport (Alcock et al., 2016). Previous studies using TatA‐YFP to monitor TatA assembly showed that a Gln8Ala substitution does not alter the wild‐type TatA assembly behavior when TatE is present (Alcock et al., 2016) (confirmed in Figure 2). Whether this is also true in the absence of TatE could not be determined using the imaging assay because TatA‐YFP itself becomes trapped in the assembled state under these conditions (Leake et al., 2008) (Figure 2). Using the direct solubilization assay, we now find that when TatE is present the TatA^Q8A^ variant phenocopies the wild‐type protein, as expected from the imaging assay, but in the absence of TatE the TatA^Q8A^ variant exhibits substrate overproduction‐independent but protonophore‐sensitive TatA‐TatC interactions (Figure 4, compare strains A^Q8A^BCE and A^Q8A^BC) like most of the other oligomer‐stabilized TatA variants analyzed here. Thus, TatA^Q8A^ is an additional substitution that is capable of trapping TatA in the assembled state. Control experiments in which the TatE counterpart of TatA Gln8 is inactivated through a Lys8Ala substitution confirm that the ability of TatE to suppress the phenotype of a Gln8‐substituted TatA variant depends on the equivalent residue in TatE (Figure S1).

Intriguingly, the direct solubilization assay shows that the TatA‐YFP fusion protein phenocopies the assembly behavior of TatA^Q8A^ in showing substrate overproduction‐independent but protonophore‐sensitive TatA‐TatC interactions only when TatE is absent (Figure 4, compare strains AyBCE and AyBC). By contrast, the F39A and D31K variants of TatA show the same general pattern of behavior in the direct solubilization assay whether TatE is present or not (Figure 4 compare strains A^F39A^BCE and A^F39A^BC, A^D31K^BCE and A^D31K^BC).

Although we had anticipated that the use of trapped or constitutively assembled TatA oligomers would improve the recovery of TatABC complexes relative to the actively translocating wild‐type strain we did not find a large increase in the amount of TatA co‐immunoprecipitating with TatC when extracted from spheroplasts of these strains (Figure 4; Figure S2). Thus, other approaches will need to be found to stabilize the active translocation site for purification. It has been reported that the thylakoid Tat system can be trapped in the assembled state using truncated substrates (Aldridge et al., 2014; Asher & Theg, 2021) and this approach would be worth exploring in future work with the bacterial system.

## 3 DISCUSSION

The active Tat translocon is formed transiently during the Tat transport cycle by the PMF‐dependent oligomerization of TatA on to a TatABC core. Characterization of this complex is clearly key to elucidating the mechanism of Tat transport. However, the complex has so far proven refractory to isolation. In this work, we show that solubilizing the assembled translocation complex directly from energized membranes leads to the recovery of much higher levels of TatA with TatBC thanthat observed when conventional membrane preparations are used. This improvement in the retention of translocation site integrity was not by itself sufficient to allow the isolation of assembled translocation sites in high yield because most of the TatA molecules present in the cells were still not recovered in complex with TatBC. Nevertheless, the new solubilization strategy provides an important step towards the goal of purifying intact translocation sites.

Our detergent solubilization method relies on the energization of the starting membrane system. This is demonstrated by the failure to recover assembled translocon complexes in control experiments where the membranes are first de‐energized, either by mechanical rupture or by protonophore treatment (Figure 1a,b). Because detergent solubilization of the spheroplast membrane will itself abolish the transmembrane PMF, the trapping of assembled translocon complexes in our experiments suggests that complex disassembly following loss of the PMF is slower than extraction of the complex into micelles.

Very recently, Asher and Theg reported that TatA binding to the chloroplast TatBC complex could be detected by blue native PAGE following detergent solubilization of thylakoids that had been incubated with a truncated substrate protein (Asher & Theg, 2021). Although the thylakoids in this experiment were not actively energized at the point of detergent addition, it is plausible that the complex identified by Asher and Theg is analogous to the substrate‐ and PMF‐dependent TatABC complexes extracted from spheroplasts in our direct solubilization approach.

Before the current work, the formation of the assembled Tat translocon could be detected in two ways. First, the association of TatA with TatBC can be identified through crosslinking. This approach has been widely employed in the thylakoid Tat system (e.g. Dabney‐Smith et al., 2006; Mori & Cline, 2002). However, in the bacterial system, the presence of TatA constitutively bound to TatBC makes interpretation of crosslinking more difficult. Thus, while it has been possible in the bacterial system to demonstrate the PMF‐driven movement of TatA into the vicinity of the substrate protein using photoaffinity crosslinking (e.g. Alami et al., 2003) and link changes in disulfide crosslinking patterns to transport‐permissive conditions (Habersetzer et al., 2017), it is not known whether this represents TatA oligomerization or movement of the constitutive TatA molecules. Our direct solubilization method avoids this ambiguity because it measures the amount of TatA associated with TatC. Second, Tat translocon assembly in bacteria can be inferred by determining the TatA oligomeric state through in‐cell fluorescence imaging. There have been concerns that the TatA‐fluorescent protein fusion proteins used in this imaging assay might not faithfully mimic the behavior of the native TatA protein because the fusions are only able to mediate Tat transport in the presence of either wild type TatA or the TatA paralogue TatE (Alcock et al., 2013; Rose et al., 2013). In the work described here, we have combined our direct solubilization approach with a co‐immunoprecipitation analysis to produce a biochemical assessment of the assembly state of the Tat translocation site that does not use a TatA fusion protein. Importantly, parallel analysis of cells by both our biochemical assay and the established imaging method reveals that the TatA‐fluorescent protein fusion recapitulates the assembly behavior of wild type TatA, at least when TatE is also present. Thus, our new biochemical assay not only provides a methodologically independent measure of Tat active site assembly but also validates the previously developed imaging assay. It is also worth emphasizing that our direct solubilization method is carried out at native levels of expression, in contrast to almost all previous biochemical work on the bacterial Tat system, and so avoids the perturbations in Tat proteins interactions now known to arise from Tat protein overproduction (Alcock et al., 2016).

Certain amino acid substitutions in the Tat components trap TatA in an assembled state. Several such substitutions have previously been described (Alcock et al., 2013, 2017; Huang et al., 2017; Leake et al., 2008) and two additional variants with this phenotype are identified here (TatA^Q8A^ in cells which also lack TatE, and TatC^E170A^). We explored whether substitutions of this type would assist in isolating the Tat translocon. This approach did not substantially increase the levels of TatA extracted in complex with TatBC relative to solubilization of actively translocating wild type spheroplasts. Nevertheless, these experiments did uncover differences in the behavior of the inactive variants, which provide new insight into the Tat translocation cycle.

**FIGURE 5:**
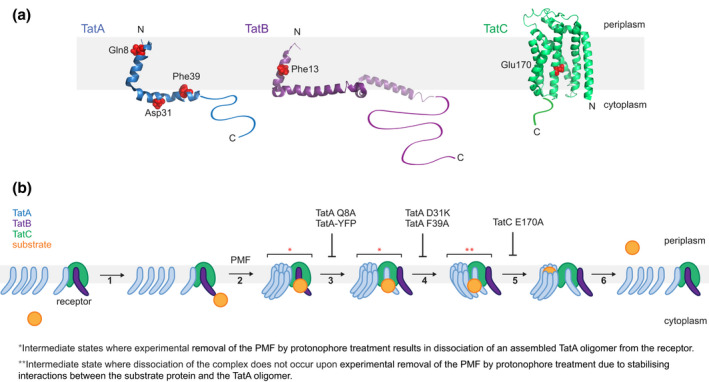
(a) Locations of amino acid substitutions in the Tat protein variants characterized in this work. The substituted amino acids are shown in red spacefilling representation on cartoon representations of the structures of the Tat proteins. Each protein has a flexible cytoplasmic tail at the C‐terminus which was not resolved in the structure and is hand‐drawn here. The structures shown for TatA (PDB 2LZR) and TatB (PDB 2MI2) are those of the proteins from *E. coli*. The structure of *Aquifex aeolicus* TatC is shown (PDB 4B4A) with the residue equivalent to *E. coli* Glu170 (*A. aeolicus* Glu165) on TM4 highlighted. (b) Schematic model of the Tat translocation cycle. The model is based on the current understanding of Tat mechanism (Palmer & Stansfeld, 2020) but modified to incorporate the results of the current study. At the start of the cycle TatB is bound at the TatC polar cluster site and the constitutively bound TatA is bound elsewhere on TatC. (1) The signal peptide of the substrate protein binds to a surface‐exposed site on the receptor complex. (2) In a PMF‐dependent process the signal peptide of the substrate protein becomes more deeply inserted into the receptor complex and a TatA oligomer is assembled on the receptor complex from TatA protomers recruited from the membrane pool. (3) TatA replaces TatB at the polar cluster site. Binding of TatA to the polar cluster site is inhibited by removal of Gln8, or by fusion to YFP, but transport can be restored by TatE. (4) The passenger domain of the substrate protein forms interactions with the cytoplasmic domain of the TatA oligomer, bridging the TatA oligomer and the receptor and stabilizing the holocomplex against removal of the PMF. This process requires TatA APH residues Asp31 and Phe39. (5) The substrate protein passenger domain is released from the receptor and transferred to the TatA oligomer in a process that requires TatC Glu170. (6) The substrate protein is transported by the TatA oligomer, the TatA oligomer disassembles, and TatB displaces TatA from the polar cluster site on TatC.

The assembly trapping amino substitutions studied here are located in all three proteins of the Tat system and in structurally diverse elements of these components (Figure 5a). TatA and TatB are homologous proteins comprising a short transmembrane helix (TMH) followed at the cytoplasmic side of the membrane by an amphipathic helix (APH) and then a natively unstructured tail (Hu et al., 2010; Rodriguez et al., 2013; Zhang et al., 2014). Trapping substitutions are found in the TMHs of both TatA (TatA Q8A) and TatB (TatB F13Y) and in the APH of TatA (TatA D31K and TatA F39A). TatC has a cupped hand‐shaped structure and the trapping substitution identified here (TatC E170A) affects the only polar residue present on the lipid‐exposed concave face of the protein (Ramasamy et al., 2013; Rollauer et al., 2012).

Our combined biochemical and imaging analysis of the assembly trapped variants revealed differences in their behavior. This indicates underlying mechanistic differences in the way these variants have been trapped and suggests that the substitutions resolve distinct substates in the Tat transport mechanism. The variants can be divided into three classes based on their assembly behavior. In the first class, represented by TatC^E170A^, the assembled state is stable to removal of the PMF. In the two remaining classes the TatA oligomer is released from TatC when the PMF is collapsed. These two classes are both comprised of TatA variants but differ in whether the TatA paralogue TatE suppresses the trapping phenotype: the class represented by TatA^D31K^ and TatA^F39A^ is largely insensitive to the presence of TatE, while the class represented by TatA^Q8A^ and the TatA‐YFP fusion traps the assemble state only when TatE is absent. In Figure 5b, we present a speculative model for the Tat translocation cycle based on these three substitution classes and on further considerations discussed below.

Our discovery that the TatA^Q8A^ and TatC^E170A^ variants are assembly‐locked means that these substitutions must block the Tat translocation cycle at a step after TatA oligomerization has occurred. This information requires the previously proposed mechanistic roles of these residues to be re‐evaluated. Following activation of the receptor complex by substrate binding, TatA is inferred to displace TatB from a polar cluster site on TatC in a process that requires residue Q8 in the TatA TMH (Alcock et al., 2016; Habersetzer et al., 2017). We previously hypothesized that the TatA molecule that is taken up into the polar cluster site nucleates the formation of the TatA oligomer (Alcock et al., 2016). However, because we now show that a TatA^Q8A^ variant is trapped in the assembled state, TatA oligomer assembly must precede TatA entry to the polar cluster site and, therefore, cannot be nucleated by this event. Consequently, we now propose that following TatA oligomer assembly a TatA protomer inserts into the polar cluster site on TatC to trigger the next step in the transport cycle. This suggestion is incorporated into our transport cycle model in Figure 5b. Because the TatA oligomer is released by protonophore treatment of the TatA^Q8A^ variant, we can infer that the connection between the TatA oligomer and the receptor complex still requires stabilization by the PMF until at least the polar cluster exchange step in the cycle. Residue E170 in TatC has previously been inferred to interact with either the substrate signal peptide or with TatA (Aldridge et al., 2014; Berks et al., 2014; Blummel et al., 2017; Ramasamy et al., 2013; Rollauer et al., 2012). These proposals are not invalidated by our observation that a TatC^E170A^ variant is assembly‐locked. Nevertheless, our data indicate that E170 is not involved in either the initial steps of signal peptide binding (as also suggested by the data of Holzapfel et al., 2007, Ma & Cline, 2013) or TatA oligomerization. TatC^E170A^ is the only one of the assembly‐locked variants in which the TatA oligomer fails to dissociate from TatC upon dissipation of the PMF suggesting that the translocon is stabilized by additional interactions in the E170A‐trapped state. One possible scenario is that TatC^E170A^ is compromised in the release of substrate from the receptor complex to the TatA oligomer for transport and that the substrate is simultaneously bound, and thus bridges, both components as illustrated in Figure 5b.

In contrast to the TatA APH substitutions, the Q8A variant only traps the Tat system in an assembled state if the TatA paralog TatE is absent or has the equivalent polar residue substituted (Figure 4; Figure S1). Given the low cellular concentration of TatE relative to TatA (Jack et al., 2001), these observations are consistent with Gln8 functioning solely through its interaction with the TatC polar cluster site, since binding of a single TatE molecule at this site would be sufficient to support the function of multiple TatA Q8A protomers in the main part of the TatA oligomer that are not directly bound to the site. By contrast, the residues substituted in the inactive APH variants would be functionally important in all TatA protomers in the assembled translocon.

A key observation in this study is that the TatABC complexes trapped by mutation do not respond to removal of the PMF in the same way as substrate‐assembled complexes. In the case of the substrate‐assembled wild‐type complexes, collapsing the PMF results both in TatA oligomer disassembly, as assessed by imaging experiments, and the loss of TatA‐TatC interactions, as judged by our solubilization assay. This behavior is in accord with the current model for Tat transport in which the oligomerization of TatA only occurs when TatA is associated with the TatBC complex. With the mutant‐trapped complexes, by contrast, TatA oligomers are still observed by imaging after the PMF has been removed even though (with the exception of the TatC^E170A^ variant) the biochemically monitored TatA‐TatC interaction is lost. The most straightforward interpretation of this behavior is that the TatA oligomer has become detached from the receptor complex. This finding is difficult to reconcile with mechanistic models in which the TatA oligomer is assembled internally within the receptor complex (Blummel et al., 2015; Frobel et al., 2019) but instead provides strong support for models in which the TatA oligomer assembles on the periphery of the receptor complex (Tarry et al., 2009; Figure 5b). In such a model, the receptor‐bound signal peptide might extend through an opening in the wall of the receptor complex to allow the substrate passenger domain to access the peripherally‐associated TatA oligomer. Our observations also lead to the surprising conclusion the TatA oligomer is stable in the absence of the receptor complex that triggers its formation, in contrast to the current model for Tat transport where TatA oligomer stability depends on remaining associated with the receptor complex.

We cannot at this point exclude the alternative possibility that in the trapped mutants TatA remains in complex with TatBC but that removing the PMF alters the interactions between the two components in a way that renders the interaction interface labile to detergent. Nor can we completely exclude the possibility that the imaging and solubilization assays are characterizing two different Tat protein populations in the mutant strains. Attempts to distinguish between these models by assessing co‐localization of fluorescent protein fusions of TatA with TatB or TatC in live cells proved to be unfeasible because strains combining the two types of fusion protein failed to support Tat transport activity (S.Hickman and B.C.Berks unpublished observations). It is unlikely that the protonophore‐resistant TatA oligomers are directly stabilized by the amino acid substitutions that were engineered into the TatA protein since the same phenotype is seen with a substitution that is not in TatA (TatB F13Y). We emphasize that our conclusion that trapping mutations fall into distinct functional classes that affect different mechanistic substeps during the assembled state follows from our data irrespective of any interpretation of how these substitutions are working at the molecular level or what exactly the different assays show.

In conclusion, the work described here has started to resolve distinct mechanistic steps in the Tat translocation cycle that take place following TatA oligomerization on to the receptor complex and suggests a less intimate connection between the TatA oligomer and the receptor complex than assumed in current mechanistic models.

## 4 EXPERIMENTAL PROCEDURES

### 4.1 Strain and plasmid construction

The plasmids and strains used in this work are listed in Tables 2 and 3.

**TABLE 2:** Plasmids used in this study

Plasmid name	Abbreviation	Description	Reference
pQE80‐CueO		Synthesis of *E. coli* CueO with a C‐terminal His_6_ tag	Leake et al. (2008)
p101C*TatBC	pBC	Very low copy vector for expression of *tatBC* from the *tatA* promoter with a modified RBS	Alcock et al. (2013)
p101C*BC FEA	pBC^FEA^	pBC, with *F94A, E103A* and *E170A* mutations in *tatC*	Alcock et al. (2013)
p101C*BC E170A	pBC^E170A^	p101C*TatBC with *E170A* mutation in *tatC*	This study
p101C*BC FEAEA	pBC^FEA,E170A^	p101C*BC with *F94A, E103A* and *E170A* mutations in *tatC*	This study
p101C*BC F13Y	pBC^F13Y^	p101C*TatBC with *F13Y* mutation in *tatB*	Huang et al. (2017)
p101C*BC FYFEA	pBC^FEA,F13Y^	p101C*TatBC with *F13Y* mutation in *tatB* and *F94A* and *E103A* mutations in *tatC*	This study
p101CEPE	pE	Very low copy vector for expression of *tatE* from its own promoter	This study
p101CEPE K8A	pE^K8A^	p101CEPE with *K8A* mutation in *tatE*	This study
pGEMT‐easy		TA cloning vector	Promega
pTatBC101		Very low copy pTH19Cr backbone used for construction of p101CEPE and p101CEPE K8A	Alcock et al. (2013)
pKSUniA		pBluescript‐based vector carrying P_*tatA*_ *‐tatA*	Koch et al. (2012)
pBSTatAry		pBluescript‐based vector carrying P_*tatA*_ *‐tatA‐EAK‐eyfp* ^*A206K*^	Alcock et al. (2013)
pRS552		Shuttle vector for integration of DNA at the *E. coli* phage lambda attachment site (*attB*)	Simons et al. (1987)

**TABLE 3:** Strains used in this study

Strain name	Abbreviation	Genotype	Reference
MC4100	ABCE	F‐, Δ*lacU169, araD139, rpsL150, relA1, ptsF, rbsR, flbB5301*	Casadaban and Cohen (1979)
DADE		MC4100 Δ*tatABC* Δ*tatE*	Wexler et al. (2000)
ELV16	BC	MC4100 Δ*tatA* Δ*tatE*	Sargent et al. (1999)
MΔBC	AE	MC4100 Δ*tatBC*	Alcock et al. (2013)
ELV16 λAry	AyBCE	MC4100 Δ*tatA, attB::*P_*tatA*_ *tatA‐EAK‐eyfp* ^*A206K*^ (kan_r_)	Alcock et al. (2013)
MΔABC λAry	AyE	MC4100 Δ*tatABC::apra, attB::*P_*tatA*_ *tatA‐EAK‐eyfp* ^*A206K*^ (kan^r^)	Alcock et al. (2017)
J1M1	ABC	MC4100 Δ*tatE*	Sargent et al. (1998)
JARV16 λAry	AyBC	MC4100 Δ*tatA* Δ*tatE, attB::*P_*tatA*_ *tatA‐EAK‐eyfp* ^*A206K*^ (kan^r^)	Leake et al. (2008)
DADE λAry	Ay	MC4100 Δ*tatABC* Δ*tatE, attB::*P_*tatA*_ *tatA‐EAK‐eyfp* ^*A206K*^ (kan^r^)	This study
ELV16 λA F39A	A^F39A^BCE	MC4100 Δ*tatA, attB::*P_*tatA*_ *tatA* ^*F39A*^ (kan^r^)	Alcock et al. (2013)
ELV16 λAry F39A	Ay^F39A^BCE	MC4100 Δ*tatA, attB::*P_*tatA*_ *tatA* ^*F39A*^ *‐EAK‐eyfp* ^*A206K*^ (kan^r^)	Alcock et al. (2013)
JARV16 λA F39A	A^F39A^BC	MC4100 Δ*tatA* Δ*tatE, attB::*P_*tatA*_ *tatA* ^*F39A*^ (kan^r^)	Alcock et al. (2013)
MΔABC λAry F39A	Ay^F39A^E	MC4100 Δ*tatABC::apra, attB::*P_*tatA*_ *tatA* ^*F39A*^ *‐EAK‐eyfp* ^*A206K*^ (kan^r^)	This study
DADE λAry F39A	Ay^F39A^	MC4100 Δ*tatABC* Δ*tatE, attB::*P_*tatA*_ *tatA* ^*F39A*^ *‐EAK‐eyfp* ^*A206K*^ (kan^r^)	Alcock et al. (2013)
ELV16 λA D31K	A^D31K^BCE	MC4100 Δ*tatA, attB::*P_*tatA*_ *tatA* ^*D31K*^ (kan^r^)	This study
ELV16 λAry D31K	Ay^D31K^BCE	MC4100 Δ*tatA, attB::*P_*tatA*_ *tatA* ^*D31K*^ *‐EAK‐eyfp* ^*A206K*^ (kan^r^)	Alcock et al. (2017)
JARV16 λA D31K	A^D31K^BC	MC4100 Δ*tatA* Δ*tatE, attB::*P_*tatA*_ *tatA* ^*D31K*^ (kan^r^)	Alcock et al. (2017)
DADE λAry D31K	Ay^D31K^	MC4100 Δ*tatABC* Δ*tatE, attB::*P_*tatA*_ *tatA* ^*D31K*^ *‐EAK‐eyfp* ^*A206K*^ (kan^r^)	Alcock et al. (2017)
MΔABC λAry D31K	Ay^D31K^E	MC4100 Δ*tatABC::apra, attB::*P_*tatA*_ *tatA* ^*D31K*^ *‐EAK‐eyfp* ^*A206K*^ (kan^r^)	This study
ELV16 λA Q8A	A^Q8A^BCE	MC4100 Δ*tatA, attB::*P_*tatA*_ *tatA* ^*Q8A*^ (kan^r^)	Alcock et al. (2016)
ELV16 λAry Q8A	Ay^Q8A^BCE	MC4100 Δ*tatA, attB::*P_*tatA*_ *tatA* ^*Q8A*^ *‐EAK‐eyfp* ^*A206K*^ (kan^r^)	Alcock et al. (2016)
JARV16 λA Q8A	A^Q8A^BC	MC4100 Δ*tatA* Δ*tatE, attB::*P_*tatA*_ *tatA* ^*Q8A*^ (kan^r^)	Alcock et al. (2016)
JARV16 λAry Q8A	Ay^Q8A^BC	MC4100 Δ*tatA* Δ*tatE, attB::*P_*tatA*_ *tatA* ^*Q8A*^ *‐EAK‐eyfp* ^*A206K*^ (kan^r^)	Alcock et al. (2016)
DADE λAry Q8A	Ay^Q8A^	MC4100 Δ*tatABC* Δ*tatE, attB::*P_*tatA*_ *tatA* ^*Q8A*^ *‐EAK‐eyfp* ^*A206K*^ (kan^r^)	This study
MΔABC λAry Q8A	Ay^Q8A^E	MC4100 Δ*tatABC::apra, attB::*P_*tatA*_ *tatA* ^*Q8A*^ *‐EAK‐eyfp* ^*A206K*^ (kan^r^)	This study

All codon changes were introduced by site‐directed mutagenesis using the Quikchange method (Agilent). For construction of strains carrying *tatA* or *tatA‐yfp* mutations, the required codon changes were first carried out in plasmid pKSUniA (Koch et al., 2012) (for *tatA*) or pBSTatAry (Alcock et al., 2013) (for *tatA‐yfp*). The mutated alleles were moved into the shuttle vector pRS552 (Simons et al., 1987) by restriction cloning with BamHI and EcoRI, and delivered onto the chromosome of the desired MC4100‐derived strain.

For construction of p101CEPE and p101CEPE K8A, P_*tatE*_ *‐tatE* was amplified from MC4100 genomic DNA by PCR with primers EcoRI‐TatEprom‐F (5′‐CCCGAATTCCAACTGCCCGTCTTAAACAAC‐3′) and PstI‐TatE‐R (5′‐CAACTGCAGTCACTCTTTATGAGAGAGCTTTTC‐3′) and cloned into pGEM‐T Easy (Promega) by TA cloning. A *lys8* to *ala* codon change was introduced by site‐directed mutagenesis and both the parental and mutant genes were excised with EcoRI and PstI and individually cloned into pTatBC101 (Alcock et al., 2013) that had been digested with the same enzymes to release the P_*tatA*_ *‐tatBC* insert.

### 4.2 Analytical methods

For co‐immunoprecipitation experiments cultures of freshly transformed cells were grown aerobically in LB medium at 37°C, harvested at mid‐log phase, and resuspended in SP buffer (50 mM Tris–HCl pH 7.6, 10% sucrose, 2 mM EDTA). Cells carrying pQE80‐CueO for production of CueO were induced with 1 mM IPTG for 1 h prior to harvesting. Cell suspensions were incubated with 0.2 mg/mL lysozyme (Sigma‐Aldrich) for 20 min at room temperature. Where indicated, 50 μM CCCP was added for the final 3 min of this incubation. 50 units/ml benzonase (Sigma‐Aldrich) was added and samples were solubilized with 1.4% (w/v) digitonin (Calbiochem) for 1 hour at room temperature. After solubilization, samples were centrifuged for 1 h at 100,000 × *g* at 4°C. Supernatants were incubated with continuous mixing with 50 μl of a 50% slurry of Ultrapure agarose beads (Life Technologies) for 20 min to absorb proteins that non‐specifically bind to the affinity matrix, and the beads were then removed by centrifugation (30s at 20,000 × *g*). TatC‐containing complexes were incubated with α‐TatC antibodies for 1.5 h at room temperature, then 20 μl of a 50% slurry of protein A sepharose (Genscript) was added, and the incubation was continued for a further 1.5 h at room temperature with continuous mixing. Unbound material was removed by centrifugation, and the protein A sepharose was washed by centrifugation with 2 × 1 ml IP buffer (10 mM Tris–HCl pH 7.6, 140 mM NaCl, 1 mM EDTA, 0.1% (w/v) digitonin). Bound proteins were then eluted in Laemmli sample buffer (Laemmli, 1970) at 55°C for 10 min. Samples were analyzed by SDS PAGE and immunoblotting.

CueO export assays were performed as previously described (Alcock et al., 2017).

SDS sensitivity was determined by dilution of overnight cultures both into LB and into LB + 4% SDS, followed by 3 h aerobic growth at 37°C. The quotient of the OD_600_ with/without SDS for the test cultures was normalized to that of the wild‐type strain.

Polyclonal antibodies against TatA, TatB, and TatC were as described previously (Alcock et al., 2016). YFP was detected using antibodies against GFP (clone JL‐8, Clontech). CueO was detected using antibodies against the C‐terminal His_6_ tag (Thermo Scientific).

Immunoprecipitation and immunoblotting data are representative of experiments carried out a minimum of three times with independent biological replicas.

### 4.3 Microscopy

Cells for fluorescence microscopy were cultured, with or without CueO overproduction, as for the co‐immunoprecipitation experiments, and prepared in tunnel slides as previously described (Alcock et al., 2013). Where indicated cells were incubated with 50 μM CCCP for 10 min prior to imaging.

Fluorescence images were acquired using a Nanoimager (Oxford Nanoimaging) equipped with a 532 nm 1 W DPSS laser, a 100x oil‐immersion objective (Olympus, numerical aperture 1.4), and an ORCA‐Flash4.0 V3 CMOS camera (Hamamatsu). Images were collected in HiLo mode (49% laser angle) at 10% laser power. For figure composition, image stacks were imported into Fiji (Schindelin et al., 2012), averaged over 60 ms and scaled to display 1400 arbitrary units (a.u.) as the maximum (white) and 550 a.u. as the minimum (black).

Fluorescence imaging data are representative of experiments carried out a minimum of three times with independent biological replicas.

Cells for light microscopy were cultured in LB to mid‐log phase, diluted, spotted onto glass slides and imaged on a phase contrast microscope with a 40x objective.

## AUTHOR CONTRIBUTIONS

**Felicity Alcock:** Conceptualization; investigation; resources; writing – original draft; writing – review and editing. **Ben C. Berks:** Conceptualization; funding acquisition; supervision; writing – original draft; writing – review and editing.

## ETHICS STATEMENT

No human or animal subjects were used in this study.

## Supporting information


Figure S1

## Data Availability

Data available on request from the authors.
