# Supplementary material for: New insights into the Tat protein transport cycle from characterizing the assembled Tat translocon
Source: Mol Microbiol. 2022 Oct 5;118(6):637–51. doi: 10.1111/mmi.14984 (PMC10092561; doi:10.1111/mmi.14984)
Supplement: Supplementary file 1 — Figure S1 [file MMI-118-637-s001.pdf]

New insights into the Tat protein transport cycle from characterising the assembled Tat translocon

Felicity Alcock[a*#] and Ben C.Berks[a#]
[a] Department of Biochemistry, University of Oxford, South Parks Road, Oxford, United Kingdom.

Running title: Analysis of the assembled Tat translocon

[#] Address correspondence to F.Alcock (felicity.alcock@newcastle.ac.uk); B.C.Berks (ben.berks@bioch.ox.ac.uk).
* Present address: Microbes in Health and Disease Theme, Newcastle University Biosciences Institute, Newcastle University, Newcastle upon Tyne, United Kingdom.

Supplementary Information

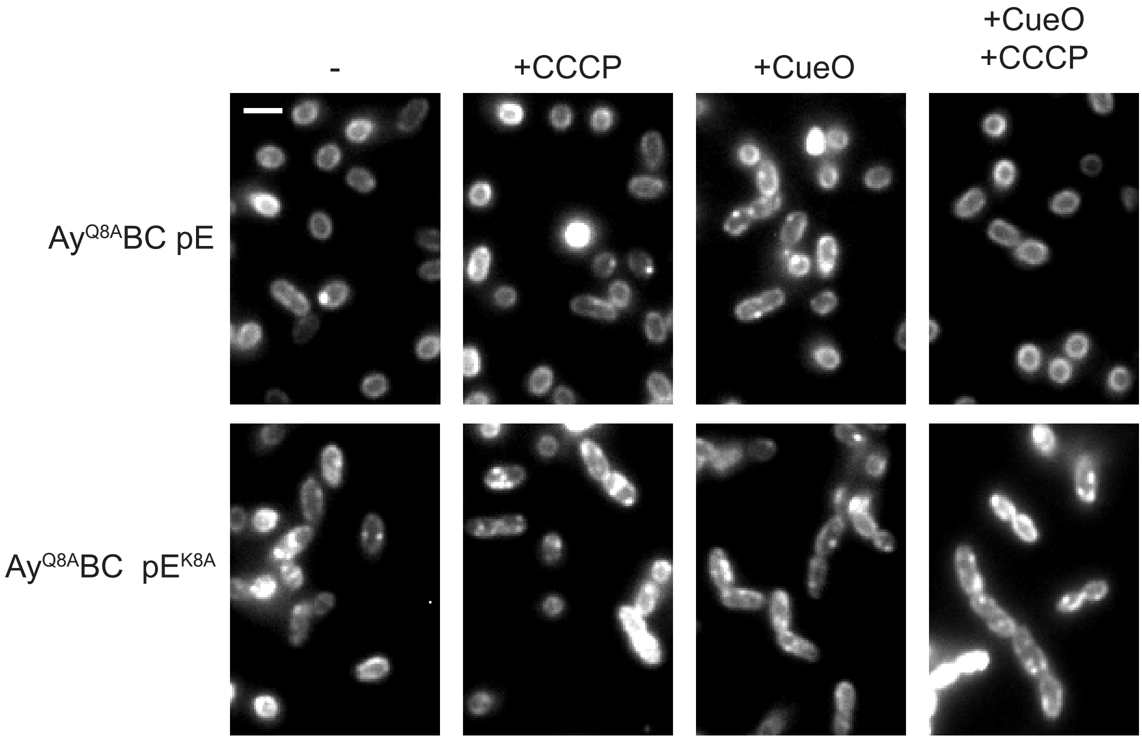

Figure S1

Effect of substituting the TatE polar cluster residue Lys8 on the substrate-induced oligomerization of TatA in live cells. Representative fluorescence images of TatA-YFP in live cells. Where indicated, strains were induced for high level production of the Tat substrate protein CueO (+CueO columns). Where specified, cells were treated with CCCP prior to imaging to dissipate the PMF (+CCCP columns). Scale bar = 2μM (shown in top left panel). Both strains express a TatA-YFP fusion containing a Q8A substitution together with TatB and TatC. They also carry a plasmid expressing either TatE (in strain $Ay^{Q8A}BC$ pE) or TatE K8A (in strain $Ay^{Q8A}BC$ pE^{K8A}).

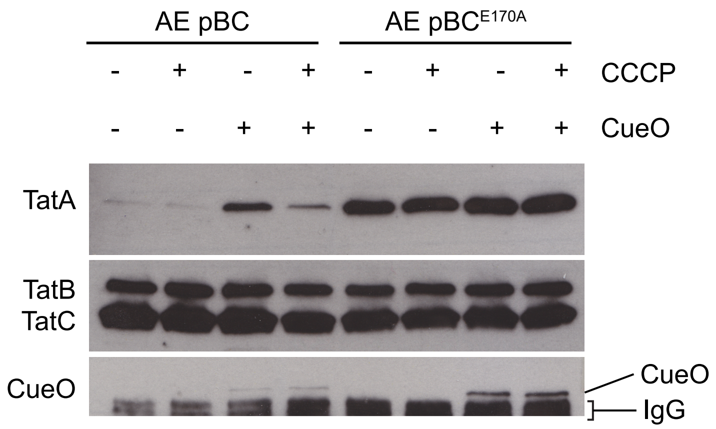

Figure S2

Comparison of TatA co-immunoprecipitation with wild-type or E170A variant of TatC. Digitionin-solubilized spheroplasts of the indicated strains were immunoprecipitated with antibodies against TatC and then immunoblotted with TatA antibodies. Where indicated, strains were induced for high level production of the plasmid-encoded Tat substrate protein CueO. Where specified, cells were treated with CCCP to dissipate the PMF before addition of detergent. Immunoprecipitated material was analysed by immunoblotting with antibodies against TatA, TatB and TatC or CueO. Elements of this figure are reproduced in Figure 1C and Figure 4. The complete experiment is shown here. Strains are Δ*tatBC* but complemented with a plasmid expressing either *tatBC* or *tatBC*^{E170A} at native levels (strains AE pBC and AE pBC^{E170A} respectively).
